# Rotablator Burr Disconnection: Can This Rare Complication Be Predicted and How to Manage?

**DOI:** 10.1016/j.jaccas.2025.105159

**Published:** 2025-08-27

**Authors:** Kevin Hamzaraj, Rayyan Hemetsberger

**Affiliations:** Department of Cardiology, Internal Medicine II, Medical University of Vienna, Vienna, Austria

**Keywords:** cardiovascular disease, complication, coronary angiography, myocardial ischemia, myocardial revascularization, percutaneous coronary intervention, stents, treatment

**Figure:**
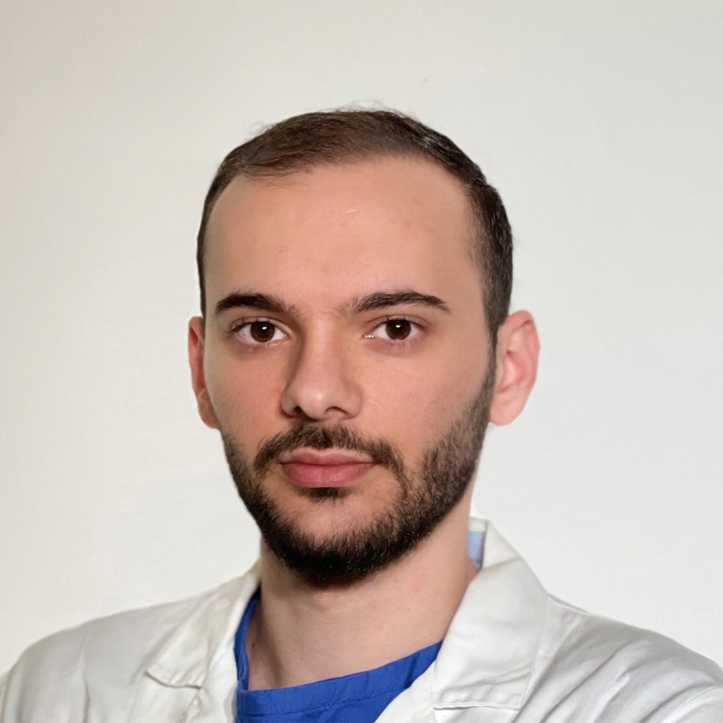

Rotational atherectomy (RA) remains a valuable tool in interventional cardiology when dealing with severely calcified coronary lesions. However, being a complex technique, in some cases the benefits of RA are accompanied by a spectrum of complications. In this issue of *JACC: Case Reports*, Yamaji et al^1^ present a valuable case series exploring one rare complication of rotational atherectomy: burr disconnection during RA procedures, observed in 6 patients over a 16-year period at a single center in Japan. To our knowledge, this is the largest single-center experience to date dedicated to this specific complication, offering insights into mechanisms, procedural context, and prevention strategies.

The reported incidence of burr disconnection was 0.4%. Burr disconnections occurred during in-stent ablation in 4 of 6 cases, and 2 of them had an in-stent bending. Notably, in 2 patients, the complication was followed by guidewire fracture, necessitating emergency coronary artery bypass surgery. Although rare, the potential severity of this complication warrants systematic preventive efforts and clearly defined bailout strategies.

Yamaji et al^1^ present an intriguing fluoroscopic finding: the “blank sign,” which appears as a gap between the burr and the drive shaft, typically appearing just before disconnection. This observation adds valuable insight into the mechanism of burr dislodgment. Although its detection requires meticulous, frame-by-frame fluoroscopic analysis—a task not easily achieved during time-pressured interventions—the mere knowledge of the blank sign could sharpen our vigilance. Nevertheless, once familiar with the phenomenon described by the authors, operators might begin to recognize it in real time, potentially allowing for timely intervention before disconnection occurs.

A notable procedural characteristic in this series is the use of relatively large burrs—2.15 mm or 2.25 mm in 3 of 6 cases. In contrast, current practice in the United States and Europe generally favors smaller burrs, with sizes beyond 2.0 mm now reserved for carefully selected cases. This reflects a broader shift in strategy: whereas larger burrs were more commonly used in previous years,^2^ contemporary practice has evolved alongside the development of balloon-based therapies. Today, high procedural success can often be achieved using rotational atherectomy in combination with modern, high-performance balloons, reducing the need for aggressive debulking. In Europe, this shift is reflected in the predominance of 1.5-mm and 1.75-mm burrs. In the Asia–Pacific region, however, there appears to be a greater tendency toward the use of larger burrs. This preference may partially account for mechanical complications such as burr detachment, particularly when compounded by anatomical challenges such as stent ablation, lesion angulation, and lack of coaxial alignment. Although the sample size is limited, these procedural patterns recur in the presented cases. Current expert consensus emphasizes the importance of proper case selection, careful burr manipulation using the pecking motion, correct rotational speed, avoidance of re-rotation during entrapment, and the use of guide extension catheters to facilitate burr advancement.^3^^,^^4^ In several cases in this series, these key preventive measures could have been applied. At the same time, the series reflects a gradual refinement in practice and complication management over time. Procedural standardization, in both equipment choice and technique, remains essential to minimizing such adverse events. In addition to prevention, the management of burr disconnection or entrapment requires a structured and well-practiced bailout strategy.

Drawing from both the Yamaji et al series and the recent case report by Boueri et al^5^ describing a successful “crushing technique” bailout, we propose the following simple algorithm for managing detached burrs (Figure 1):

1. If the RotaWire remains intact, attempt gentle retraction to retrieve the burr. As the spring tip of the RotaWire is thicker than the wire body, it may lodge behind the burr, facilitating its withdrawal.
2. If traction fails or the RotaWire is broken, cut the drive shaft and remove its external sheath to create additional space within the guiding catheter. Then insert a guide extension catheter and advance it along the remaining drive shaft toward the detached burr. Once the guide extension reaches the burr, remove the broken drive shaft, rewire alongside the detached burr, and attempt retrieval—either using a snare or using a partially inflated balloon positioned distal to the burr. Carefully pull the burr into the tip of the guide extension, and then withdraw the entire system en bloc into the guiding catheter. Alternatively, a ping-pong guiding catheter setup may be considered in place of a guide extension.
3. If retrieval is unsuccessful, crushing the burr with a stent may serve as a last resort—in other words, *bury the burr* against the vessel wall. Ensure full lesion coverage, and perform adequate post-dilatation.
4. If percutaneous options fail or are not feasible, surgical removal remains the final fallback.

**Figure 1:**
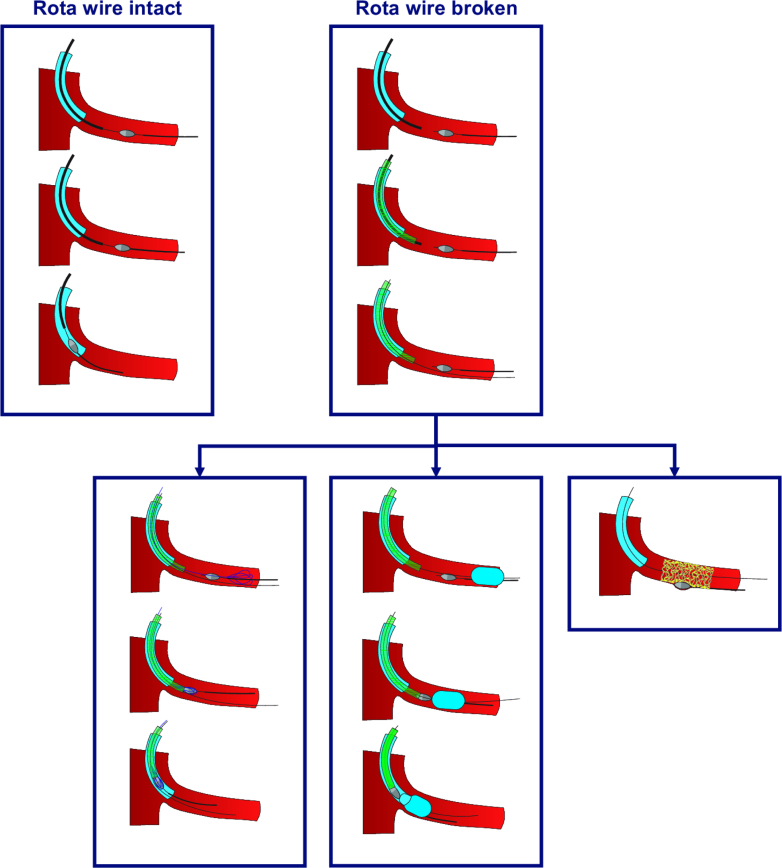
Suggested Management Algorithm for Detached Rotational Atherectomy Burrs If the RotaWire is intact, the burr may be retrieved by pulling on the wire. The spring tip of the RotaWire is thicker than the wire body and may lodge behind the burr, thereby facilitating its withdrawal. If the RotaWire is broken, a guide extension catheter should be advanced over the remaining drive shaft toward the detached burr. Once the guide extension reaches the burr, the broken drive shaft is removed. The lesion is then rewired alongside the detached burr, and retrieval is attempted using either a snare or a partially inflated balloon positioned distal to the burr. The burr is then carefully pulled into the tip of the guide extension catheter, and the entire system is withdrawn en bloc into the guiding catheter. As an alternative, a ping-pong guiding catheter setup may be used in place of the guide extension. If retrieval fails, the burr may be crushed and buried against the vessel wall using a stent as a last resort.

Indeed, the rarity of this event—only 6 cases in 1,380 procedures—reaffirms the overall safety of rotational atherectomy when performed with precision and caution. Still, interventionalists should familiarize themselves with both the signs of forthcoming complication and the complete toolbox of retrieval strategies, including those that may require immediate escalation. The report by Yamaji et al is a valuable addition to the literature on RA-related complications. Whereas the blank sign may be more of a retrospective clue than a real-time warning, these cases underscore the need for procedural vigilance, appropriate equipment use, and advance planning. Although the mechanisms of burr detachment are not yet fully understood, a structured approach to management may help operators respond effectively and mitigate clinical risk.

## Funding Support and Author Disclosures

Dr Hemetsberger has received speakers' honoraria from Boston Scientific, Biotronik, Abbott, Abiomed, Shockwave, Cordis, and B. Braun outside the present work and institutional research grants from Boston Scientific, Abbott, and Cordis. Dr. Hamzaraj has reported that he has no relationships relevant to the contents of this paper to disclose.
